# Removal of Ag remanence and improvement in structural attributes of silicon nanowires array via sintering

**DOI:** 10.1038/s41598-021-03654-5

**Published:** 2021-12-17

**Authors:** Paresh Kale, Mihir Kumar Sahoo

**Affiliations:** 1 Department of Electrical Engineering, NIT Rourkela, Odisha, 769008 India; 2 DST-IIT Bombay Energy Storage Platform On Hydrogen, IIT Bombay, Maharashtra, 410076 India

**Keywords:** Materials for devices, Materials for energy and catalysis, Nanoscale materials

## Abstract

Metal-assisted chemical etching (MACE) is popular due to the large-area fabrication of silicon nanowires (SiNWs) exhibiting a high aspect ratio at a low cost. The remanence of metal, i.e., silver nanoparticles (AgNPs) used in the MACE, deteriorates the device (especially solar cell) performance by acting as a defect center. The superhydrophobic behavior of nanowires (NWs) array prohibits any liquid-based solution (i.e., thorough cleaning with HNO_3_ solution) from removing the AgNPs. Thermal treatment of NWs is an alternative approach to reduce the Ag remanence. Sintering temperature variation is chosen between the melting temperature of bulk-Ag (962 °C) and bulk-Si (1412 °C) to reduce the Ag particles and improve the crystallinity of the NWs. The melting point of NWs decreases due to surface melting that restricts the sintering temperature to 1200 °C. The minimum sintering temperature is set to 1000 °C to eradicate the Ag remanence. The SEM–EDS analysis is carried out to quantify the reduction in Ag remanence in the sintered NWs array. The XRD analysis is performed to study the oxides (SiO and Ag_2_O) formed in the NWs array due to the trace oxygen level in the furnace. The TG-DSC characterization is carried out to know the critical sintering temperature at which remanence of AgNPs removes without forming any oxides. The Raman analysis is studied to determine the crystallinity, strain, and size of Si nanocrystals (SiNCs) formed on the NWs surface due to sidewalls etching. An optimized polynomial equation is derived to find the SiNCs size for various sintering temperatures.

## Introduction

Derived nanostructures of silicon (Si) such as porous Si (PSi), nanowires (NWs), nanotubes, and quantum dots (QDs) are essential building blocks for various semiconductor device applications. Silicon nanowires (SiNWs) exhibit a unique quantum confinement effect (QCE) that find a wide area of applications in optoelectronics and solar cells^1–5^. The SiNWs are also helpful in energy storage^6,7^, chemical and biological sensors^8–11^, and thermoelectrics^12^. Recently, the SiNWs emerges as a powerful tool for light-harvesting antennas^13^, cellular nanobiotechnology^14^, and micro-electrochemical systems^15^.

A popular method, metal-assisted chemical etching (MACE), used for synthesizing SiNWs, fabricates a large surface area and higher aspect ratio of SiNWs array at low temperature and relatively low cost^16–19^. The metal salt (e.g., AgNO_3_, CuSO_4_) used as a catalyst in MACE plays a vital role in deciding the morphology of the SiNWs and the SiNWs based device performances. Previous study^20–22^ reported the remanence of metal particles inside the NWs array even after the thorough cleaning by HNO_3_ solution. When used in solar cells, the remanence of metal particles inside the NWs array acts as a defect center creating discrete energy levels within the forbidden energy gap, called trap level. The trap level traps the electron passing from the valence band to the conduction band and recombining with the hole (present as defects). Therefore, the complete removal of metal particles from the NWs array cut down the recombination losses to improve solar cell efficiency. However, the superhydrophobic surface of the SiNWs array disallows a liquid-based solution to remove the remanence of metal particles present at the substrate and NWs interface.

Sintering, i.e., heat treatment of SiNWs array above the melting point of the metal catalysts removes the metal particles from the NWs array without melting the NWs. The decreasing order of melting temperature of metal catalysts used in HF-containing solution is Pt (1768 °C), Cu (1084 °C), Au (1064 °C), and Ag (962 °C). The lowest melting point of Ag metal makes it suitable to be removed at a lower sintering temperature after the MACE. The Ag catalyst exhibits an added advantage of thin layer deposition, unlike Pt and Cu, forming a dense layer on the Si substrate^23^.

Reduction of size deviates drastically the behavior of nanomaterials from its bulk material. Sintering above the melting point melts the bulk materials homogeneously while the nanomaterial melts from the outer surface atoms towards the inner one (known as surface melting). The surface melting lowers the melting point of SiNWs array than the bulk-Si (1412 °C), which restricts the sintering temperature to 1200 °C. Though the melting point of AgNPs is much lesser than the bulk-Ag, a minimum sintering temperature of 1000 °C is chosen to remove the Ag remanence from the NWs array altogether. Therefore, the sintering temperature is varied from 1000 to 1200 °C to remove the Ag remanence from the SiNWs array.

**Figure 1:**
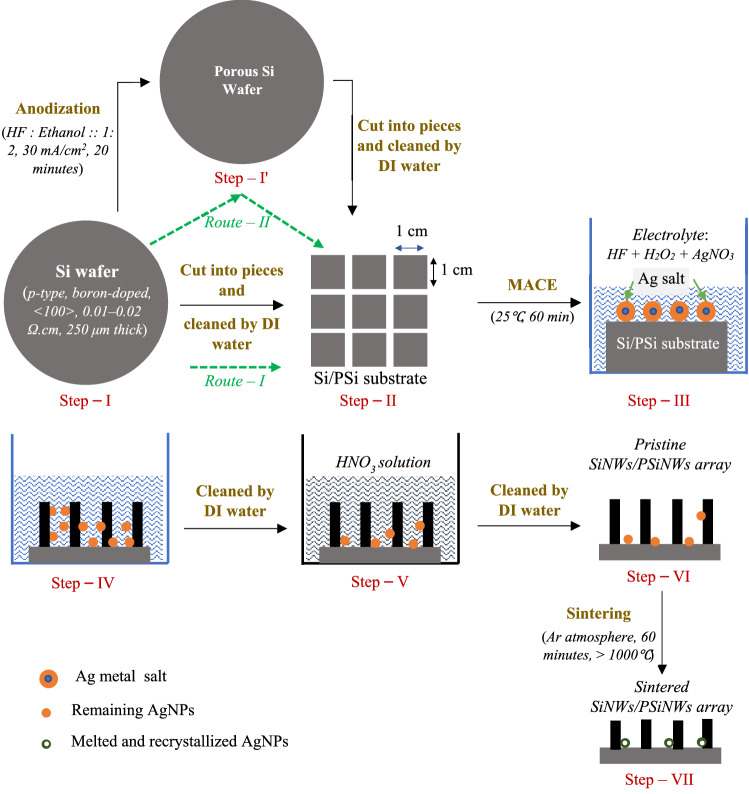
Schematic of steps involved in the fabrication and sintering of SiNWs/PSiNWs using Si/PSi substrate. The steps involved in fabricating NWs are (i) **Si/PSi substrate synthesis**: either using *Route—I* (Si wafer Si substrate) or *Route—II* (Si wafer PSi substrate, by anodization), (ii) **MACE (NWs fabrication)**: metal-assisted chemical etching of Si/PSi substrate, (iii) **Removal of Ag particles**: cleaning with HNO_3_ solution and DI water, (iv) **Sintering**: removal of remanence of AgNPs, improvement in crystallinity and strain.

The objective of the paper is to study the removal of the remanence of Ag nanoparticles (NPs) from the SiNWs array via sintering, which is a novel idea not yet reported. Additionally, sintering also helps control the morphology (crystallinity, strain, and crystal size) of the SiNWs array fabricated by MACE. Figure 1 shows steps involved in fabricating SiNWs array by MACE using Si or porous Si substrate and the sintering process to remove the Ag remanence. Primarily the Si substrate produces SiNWs array, and PSi substrate forms PSiNWs array upon MACE; therefore, both the substrates are taken into consideration to study the Ag remanence and structural attributes of the NWs.

## Results

### SEM–EDS analysis

**Figure 2:**
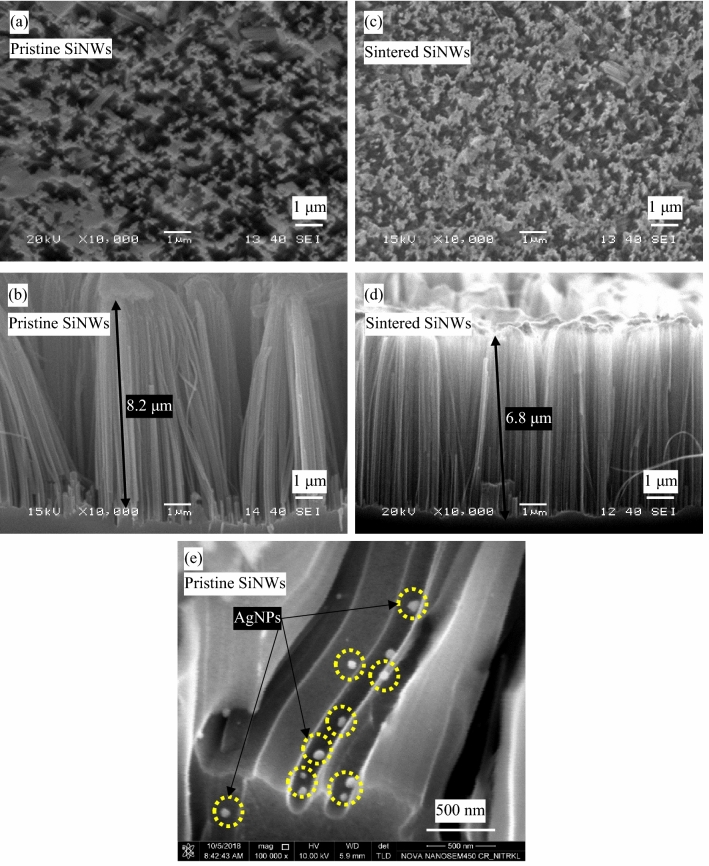
SEM image of SiNWs_0.01 sample (**a**) surface view, (**b**) cross-sectional view; and corresponding Res@1200 °C_SiNWs_0.01 (**c**) surface view, (**d**) cross-sectional view. (**e**) SEM image of SiNWs array showing the remanence of AgNPs inside the array after cleaning in HNO_3_ solution.

The agglomerated tips of the pristine NWs array, as shown in Fig. 2(a), partly become free after sintering, as shown in Fig. 2(b). The average length of the NWs array decreases from 8.2 to 6.8 µm on sintering, as shown in the cross-sectional view of Fig. 2(c, d). However, the average diameter of the pristine and sintered NWs ranges from 70 to 150 nm.

Figure 2(e) shows the remanence of AgNPs inside the NWs array after cleaning in the HNO_3_ solution, which also confirms from the EDS analysis (i.e., the presence of 0.15 at. % of Ag on the NWs array after HNO_3_ wash) in the MACE. Since the AgNPs reside on the tips of the NWs array trimmed away with the NWs on sintering, it reduces the Ag remanence to 0.03 at. % in the sintered SiNWs array. As the sintering temperature is higher than the melting point of Ag, the AgNPs present at the surface of the pristine NWs melt and evaporate, further reducing Ag remanence in the sintered NWs array.

### X-ray diffraction (XRD) analysis

**Figure 3:**
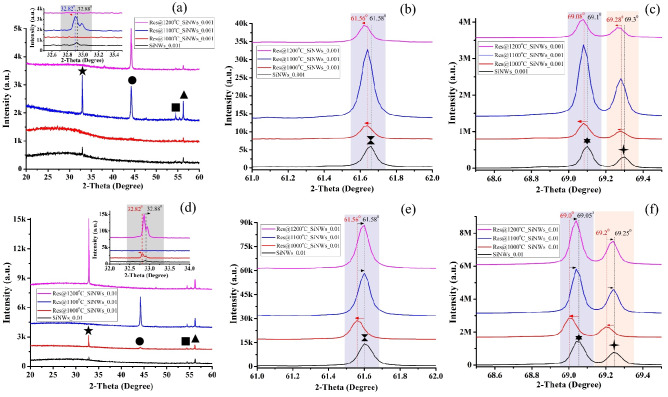
XRD pattern of pristine and sintered SiNWs_0.001 (**a**, **b**, **c**), SiNWs_0.01 (**d**, **e**, **f**). The symbol represents Filled black star (32.9°) Si (111), filled black circle (44.2°) Ag (200), filled black square (54.5°) Ag_2_O (220), filled black upward triangle (56.3°) Ag (220), black hourglass(61.6°) Si (400), (69.1°) Si (400), and (69.3°) Ag_2_O (222).

XRD analysis helps in determining the presence of AgNPs in the NWs array. Since the Ag particles oxidize quickly under atmospheric conditions^24^, at the higher sintering temperature, Ag_2_O (220) and Ag_2_O (222) planes are formed (Fig. 3), which remains for all the sintered samples at 54.5°, 69.3° respectively. The melted and evaporated Ag particles redeposit on the Si planes systematically in (200) and (111) orientations apart from (220). The most prominent XRD peak of AgNPs appears at 69.3° because of the (111) Si plane present in the NWs surface, as shown in Fig. 3(c, f).

Figure 3 shows a broad valley of XRD spectra between 2θ ranging from 20° to 30°, indicating the formation of amorphous Si and SiO_x_ in the NWs array during MACE. The broad valley of amorphous Si and SiO_x_ becomes flat in the sintered NWs array due to decreased amorphous content. The Si planes having orientation (400) (most prominent peak at 69.1°) is due to the starting Si (100) substrate, and the Si (111) plane (at 32.9°) arises because of the etching direction of Ag particles. As the doping level of starting Si/PSi substrates is high, only one Si plane, i.e., (111), is formed on the NWs surface apart from (400) plane^20^.

Figure 3(a) shows the disappearance of the Si (111) plane for sintered SiNWs at 1000 °C and 1200 °C (inset figure) and a downshift for sintered SiNWs at 1100 °C. However, the inset of Fig. 3(d) shows the disappearance of the Si (111) plane for sintered SiNWs at 1100 °C, and the peak becomes prominent at 1200 °C compared to other pristine and sintered NWs. The two XRD Si peaks (32.9° and 69.1°) shift downward for the sintered NWs at 1000 °C, indicating decreased strain on the NWs after heat treatment for all sintered NWs samples. The XRD Si peaks of sintered NWs at 1100 °C and 1200 °C either retain their position or shift upward, indicating relaxed strain or decreased lattice vibration. The XRD peak shift confirms recrystallization and changes in lattice strain of the sintered NWs. The recrystallization produces dominant Si peaks upon sintering, as shown in Fig. 3, i.e., the Si peak intensity increases. The small peak located at 61.6° is due to the residual Cu K_β_ (wavelength, *λ*_*Cuβ*_ = 0.139 nm) radiation to the Si (400) diffraction peak, as shown in Fig. 3(b, e).

### Thermogravimetry and differential scanning calorimetry (TG-DSC) analysis

**Figure 4:**
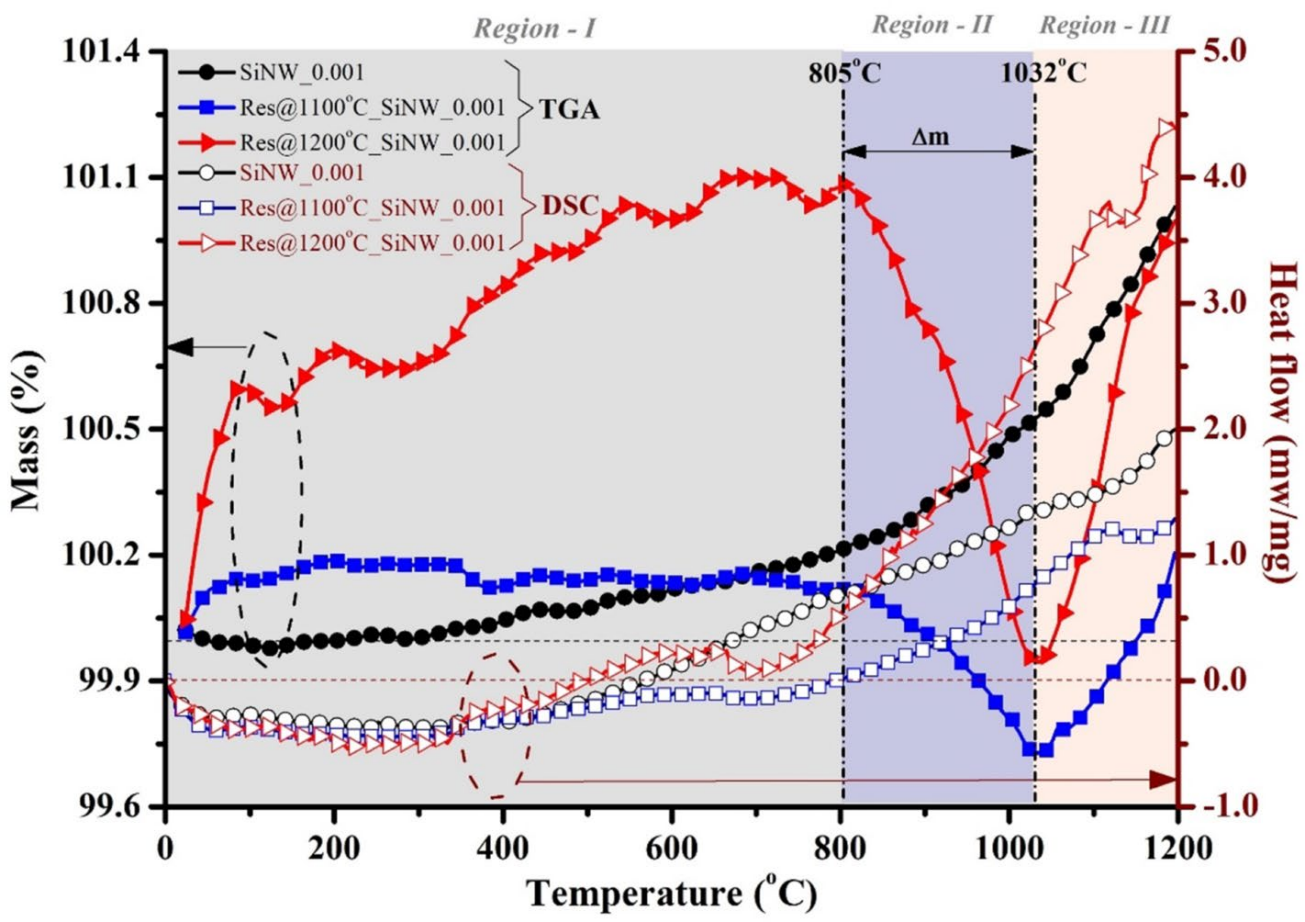
TG-DSC curve of pristine and sintered SiNWs. Thermogravimetry (TG) analysis highlights the % mass gain or loss in the heat treatment process. The differential scanning calorimetry (DSC) analysis gives heat flow, i.e., an exothermic or endothermic reaction occurs during heat treatment.

TG-DSC characterization indicates the reaction type (i.e., endothermic or exothermic) in the sintered NWs array. Although the sintering is carried out in an inert gas (Ar) atmosphere, a trace level of oxygen in the furnace reacts with Si or Ag to form respective oxides. Figure 4 shows the TG-DSC curve of the pristine and sintered SiNWs array. Heat treatment of pristine NWs increases the surface area due to merging tiny pores forming oxides (e.g., SiO and Ag_2_O) and resulting in an exothermic reaction. The exothermic reaction increases the mass (i.e., mass gain) throughout the temperature range (*Region—I* to *Region—III* of Fig. 4). However, the trapped SiO and AgNPs inside the pores converted into SiO_2_ and Ag_2_O in the sintered NWs array. Therefore, the mass gain occurs (exothermic reaction) for the sintered NWs array (*Region—I* of Fig. 4) up to 805 °C, i.e., the remaining SiO and AgNPs present after sintering gets converted to SiO_2_ and Ag_2_O.

After 805 °C, the reaction becomes endothermic up to 1032 °C due to the breaking of oxygen bonds in the SiO_2_ and Ag_2_O, resulting in a mass loss in the NWs array (*Region—II* of Fig. 4). However, above the 1032 °C, an exothermic reaction starts, i.e., oxygen reacts again with Si and AgNPs to form respective oxides (*Region—III* of Fig. 4). Therefore, the 1032 °C temperature is a critical point (i.e., critical sintering temperature, T_CS_) at which reaction type changes from endothermic to exothermic. After 805 °C, desorption and absorption of oxygen occur cyclically^25^ up to the melting point of Si (i.e., 1412 °C). As the melting point of SiNW is lower than the bulk-Si, the cyclic absorption and desorption of oxygen may last for a much lesser sintering temperature than the bulk-Si.

### Raman analysis

**Figure 5:**
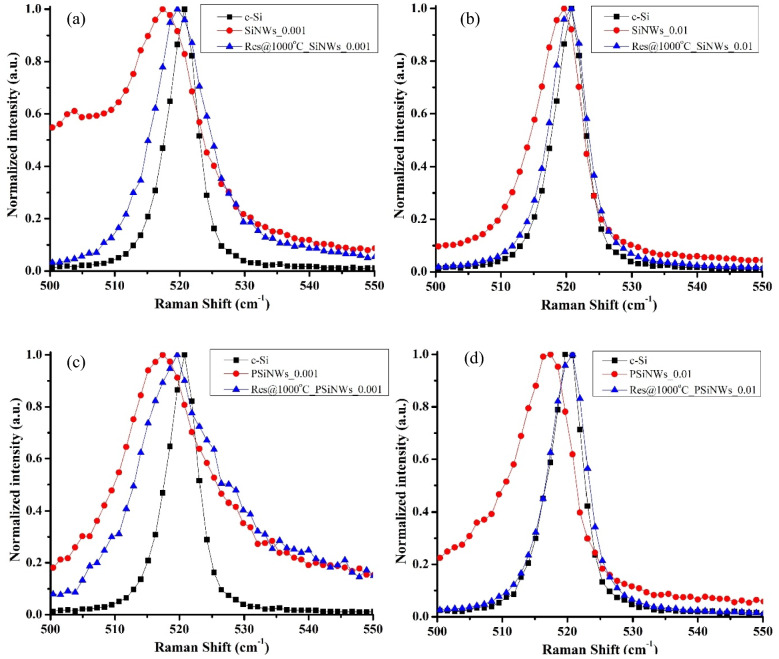
Raman spectra of pristine and sintered NWs (@1000 °C) for (**a**) SiNWs_0.001, (**b**) SiNWs_0.01, (**c**) PSiNWs_0.001, (**d**) PSiNWs_0.01.

Raman analysis estimates morphological parameters such as crystallinity, strain, and size of the Si nanocrystals formed on the NWs array. FANTUM^21^ (Fano and QCE) effect occurs for all the pristine NWs array, i.e., broadening of Raman line shape (FWHM) and shifting of Raman peak from the c-Si peak. Figure 5 shows higher FWHM of the NWs fabricated when using 0.001–0.005 Ω.cm resistivity as compared when using 0.01–0.02 Ω.cm resistivity of Si substrate, confirming the presence of higher amorphous content for lower resistivity substrate. Further, the amorphous content of NWs depends on the porosity of the sample, i.e., porous SiNWs (PSiNWs) exhibits higher FWHM compared to SiNWs sample, as shown in Fig. 5. Asymmetrical broadening of FWHM indicates the formation of the Fano effect in the NWs. The Raman spectra of pristine NWs downshifts from the c-Si peak due to QCE^21,22^ as the secondary etching or sidewalls etching creates Si nanostructures or nanocrystals on the NWs.

The FWHM of sintered NWs fabricated using lower resistive Si/PSi substrate (0.001–0.005 Ω.cm) broadens asymmetrically, confirming the retainment of the Fano effect, as shown in Fig. 5(a, c). However, the Fano effect relaxes in the sintered NWs fabricated using higher resistive Si/PSi substrate (0.01–0.02 Ω.cm), i.e., FWHM narrows down, as shown in Fig. 5(b, d). After sintering, the Raman peak shifts towards the c-Si peak, confirming recrystallization. The Raman spectra of sintered samples at 1000 °C and 1100 °C look similar (supplementary Fig. 2); however, the Raman peak downshifts again for the sintered sample at 1200 °C (supplementary Fig. 2) compared to other samples confirming an increase in QCE. Therefore, the sintered NWs fabricated using a lower resistive substrate exhibit a FANTUM effect; however, the sintered NWs fabricated using a higher resistive substrate produce only the QCE.

### Photoluminescence (PL) analysis

The PL emission determines the QCE and surface defects in the NWs array. Though the diameter of the NWs fabricated by MACE (> 70 nm) is higher than Bohr’s diameter, our previous study^22^ confirms the QCE due to secondary etching on the SiNWs’ surface. Porous Si emit blue (400 nm < λ < 500 nm), blue-green (λ ~ 490 nm), red (560 nm < λ < 860 nm), and infrared (900 nm < λ < 2000 nm) light^26^. The NWs emit red PL centered at 650 nm indicating QCE or surface/defect states. The shift in PL intensity is either due to multiple-level transition or a combined effect of temperature-assisted anti-trapping below 160 K and thermal quenching at 160–300 K. Increase in porosity red-shifts the PL from 450 to 540 nm^27^. An increase in doping concentration also red-shift the PL from 560 to 650 nm^28^. However, QCE blue-shift the PL for an increase in porosity^29^. Lin et al.^30^ reported two PL peaks at 750 nm and 850 nm, ascribed to red-excitons localized at the Si/SiO_x_ interfaces and triplet-singlet transition related to the Si–H surface states^31^.

**Figure 6:**
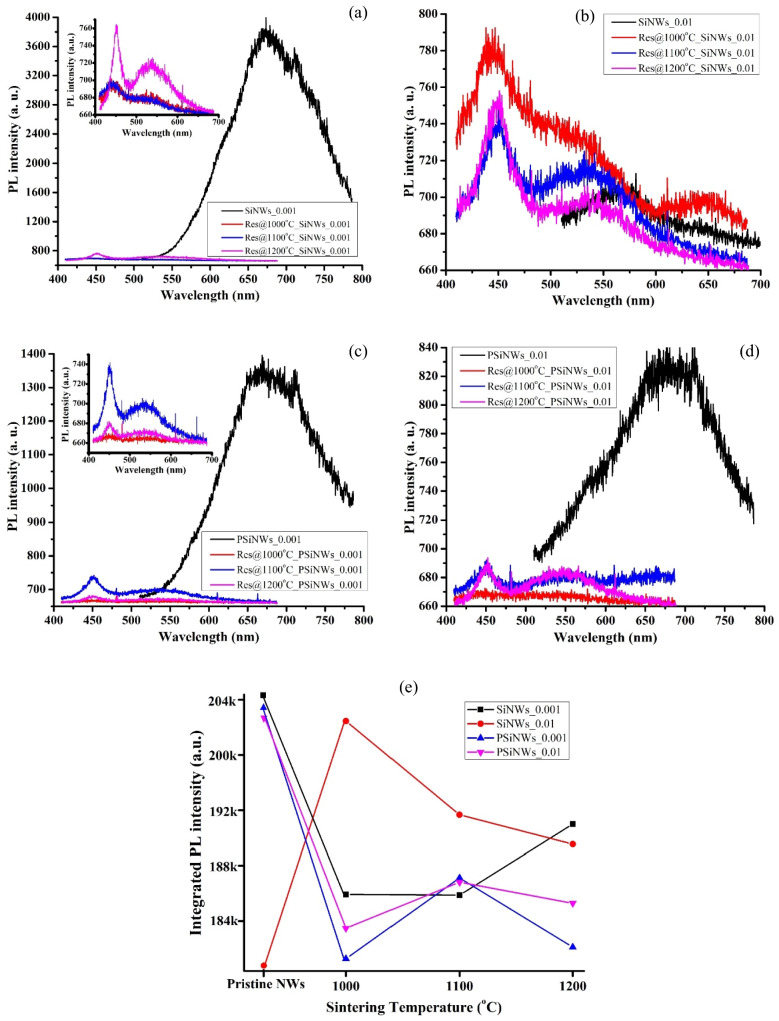
PL spectra of pristine and sintered NWs for (**a**) SiNWs_0.001, (**b**) SiNWs_0.01, (**c**) PSiNWs_0.001, (**d**) PSiNWs_0.01 sample. (**e**) integrated PL spectra of reference (i.e., pristine) and sintered NWs.

The porous structures of NWs, especially at the top part of the NWs, exhibit various energy states because of Si/SiO_x_ interfaces and QCE^22^. These defect states majorly enhance the electron–hole recombination rates and the PL emissions. The interfacial energy states of Si/SiO_x_ and Si nano-sized grains enhance the PL intensity of the pristine SiNWs. The pristine SiNWs emits a red PL centered around 675 nm (Fig. 6(a)), 575 nm (Fig. 6(b)), and that of pristine PSiNWs are 675 nm (Fig. 6(c)), 685 nm (Fig. 6(d)), respectively. The QCE of SiNWs is responsible for producing such red PL, and the secondary etching that forms nanostructures on the NWs surface originates the QCE^22^.

Sintering of NWs decreases the PL intensities drastically due to two reasons, i.e., (i) decrease in atomic % of Ag particles that reduce the hole injection (into the Si/PSi substrate) and surface oxidation and (ii) complete removal of nanostructures of Si grains and unstable Si atoms. Upon sintering, one broad PL spectra of the pristine NWs decomposes into two peaks centered around 450 nm and 550 nm. The narrow PL peak around 450 nm indicates a blue emission, and the broad PL peak around 550 nm refers to a greenish-blue light emission for the sintered NWs. Sintering blue shifts the PL spectra and produces a slight hump in the greenish-blue spectra (400 nm–600 nm). The QCE^22^ and recrystallization of the NWs responsible for the blue shift of the PL spectra. The sintered NWs exhibits lower intensity than the pristine NWs, and the integrated PL intensity (Fig. 6(e)) decreases for sintered NWs, indicating a decrease in Ag content, nanostructures of Si grains, and unstable Si atoms, which further confirms the reduction of electron–hole recombination rates and surface oxidation.

## Discussion

**Figure 7:**
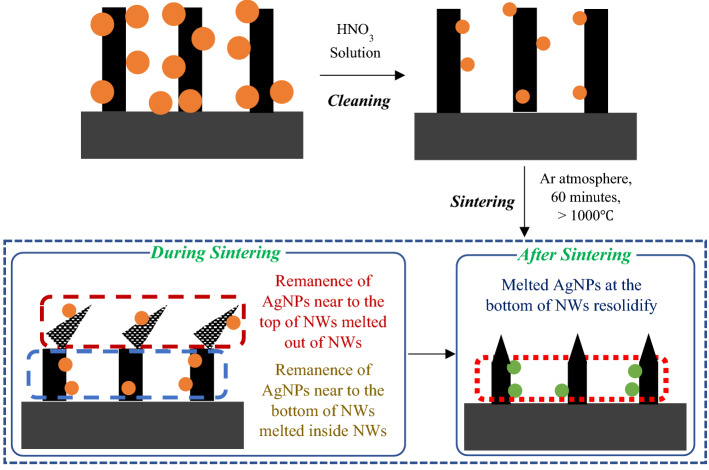
Schematic of Ag particles remanence (brown color filled circle) on the NWs after dipping in HNO_3_ solution. During sintering, the AgNPs attached to the tips of the porous NWs (white and black color pattern filled in a triangular shape) wiped away along the NWs. The AgNPs are present at the interface of the NWs base, and the Si/PSi meltdown and resolidifies after cooling.

The Ag particles act as a catalyst during the MACE and etch the Si/PSi substrate vertically to produce NWs and sidewalls of the NWs to form nanostructures on the NWs surfaces^22^. The EDS and XRD analysis reveal the Ag remanence inside the SiNWs array after thoroughly cleaning with HNO_3_ solution. Figure 7 shows the schematic of AgNPs remaining after dipping in HNO_3_ solution. The size and the atomic % of Ag reduce by dipping the NWs inside the HNO_3_ solution.

The Ag remanence on the SiNWs array act as a defect center where electron–hole recombination occurs. Apart from the defect due to the Ag remanence, the creation and migration of vacancies in the NWs array (later forms large vacant spaces) during the MACE produce defects. These defects can travel and interact freely, leading to amorphous SiNWs. Further, cleaning the Ag remanence by any liquid-based solution is impractical because the superhydrophobic SiNWs surface disallows the liquid droplet to penetrate inside the NWs array to clean the AgNPs. Removal of Ag remanence from the SiNWs array through heat treatment is an alternative approach. Sintering the SiNWs array below the melting temperature of Si (1412 °C) is an established technique to improve the morphological parameters such as crystallinity, strain, and defect sites while retaining the macroscopic shape^32–35^. Sintering at higher temperatures restricts the defects and allows spontaneous thermal vibration of Si atoms, giving rise to the recrystallization of the Si atoms. Sintering parameters such as atmosphere, time, and temperature decide the morphology of the NWs. The sintering atmosphere should be Ar or hydrogen or a mixture of both to avoid oxidation and vacancy defects in the NWs. Sintering the NWs array further reduces the Ag remanence due to two reasons, i.e., (i) trimmed NWs take away the AgNPs reside on the NWs surface, and (ii) sintering at higher temperature (> 962 °C, melting point of Ag) melts and evaporates the AgNPs. Since sintering closes the tiny pores on the NWs surface, the melted AgNPs inside the pores get trapped, which resolidifies inside the closed pores after sintering.

The thermal properties of NPs deviate from that of the bulk particles, e.g., the melting temperature of NPs is lower than that of the bulk one^36^. Nanda^37^ reviewed on size dependency melting point of NPs and reported that surface melting is an essential parameter while calculating the melting point of the NPs. The surface melting depends on the total surface energy and orientation of the NP. The surface atoms exhibit free energy, i.e., their melting point is relatively low; therefore, the AgNPs melts from the surface atom towards the inner atom, known a surface melting. During sintering the Ag atoms reorganized by decreasing the surface energy due to reduced surface area. Zhang et al.^36^ simulated spherical AgNPs having 4 nm diameter and consisting of 2123 atoms. The surface atoms began to melt at 515 °C, and the AgNPs melted completely at 827 °C. During cooling, solidification transforms the atoms to rearrange in a crystalline structure. The transformation of the FCC structure of Ag particle to HCP structure after sintering depends on the cooling procedure.

**Figure 8:**
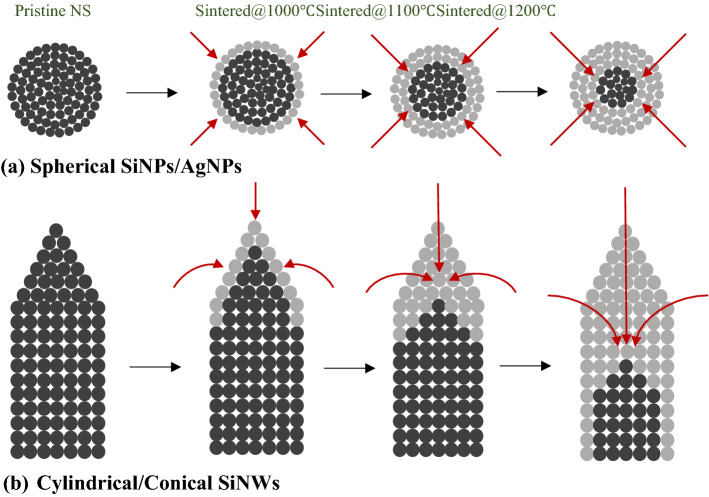
Schematic surface melting phenomena occur during sintering (**a**) spherical NPs (**b**) conical/cylindrical SiNWs. The black color-filled circle represents the atoms present in the NWs. The grey color-filled circle represents melted atoms after sintering. The dark-red color arrow indicates the surface melting path in the spherical or cylindrical nanostructures.

Figure 8(a) shows the schematic of surface melting phenomena in spherical NPs during sintering. The melting point of nanomaterials is lower than its bulk phase because bulk material melts homogeneously, and the nanomaterial melts from the outer surface atoms towards the inner surface. The nanomaterial exhibits lower melting temperature because the surface or interface atoms exhibit lower coordination (i.e., weakly bound) and lesser constrain during thermal motion. The length of the SiNWs decreases on sintering due to the surface melting, i.e., melting starts from the top to the base of the NWs array, as shown in Fig. 8(b).

Roy et al.^38^ reported Ag island growth on the oxidized Si substrate at room temperature. The crystallographic orientation of Ag nano-islands depends on the Si substrate orientation. Though the sintering was carried out in an air-tight tubular furnace, the trace level of oxygen oxidizes the Si and Ag particles to form their respective oxides, confirming the XRD and TG-DSC analysis. The oxides produce stress and strain on the NWs array. However, as observed from Fig. 3, the oxide peak intensity is low in the sintered NWs array, indicating lower oxygen content.

**Figure 9:**
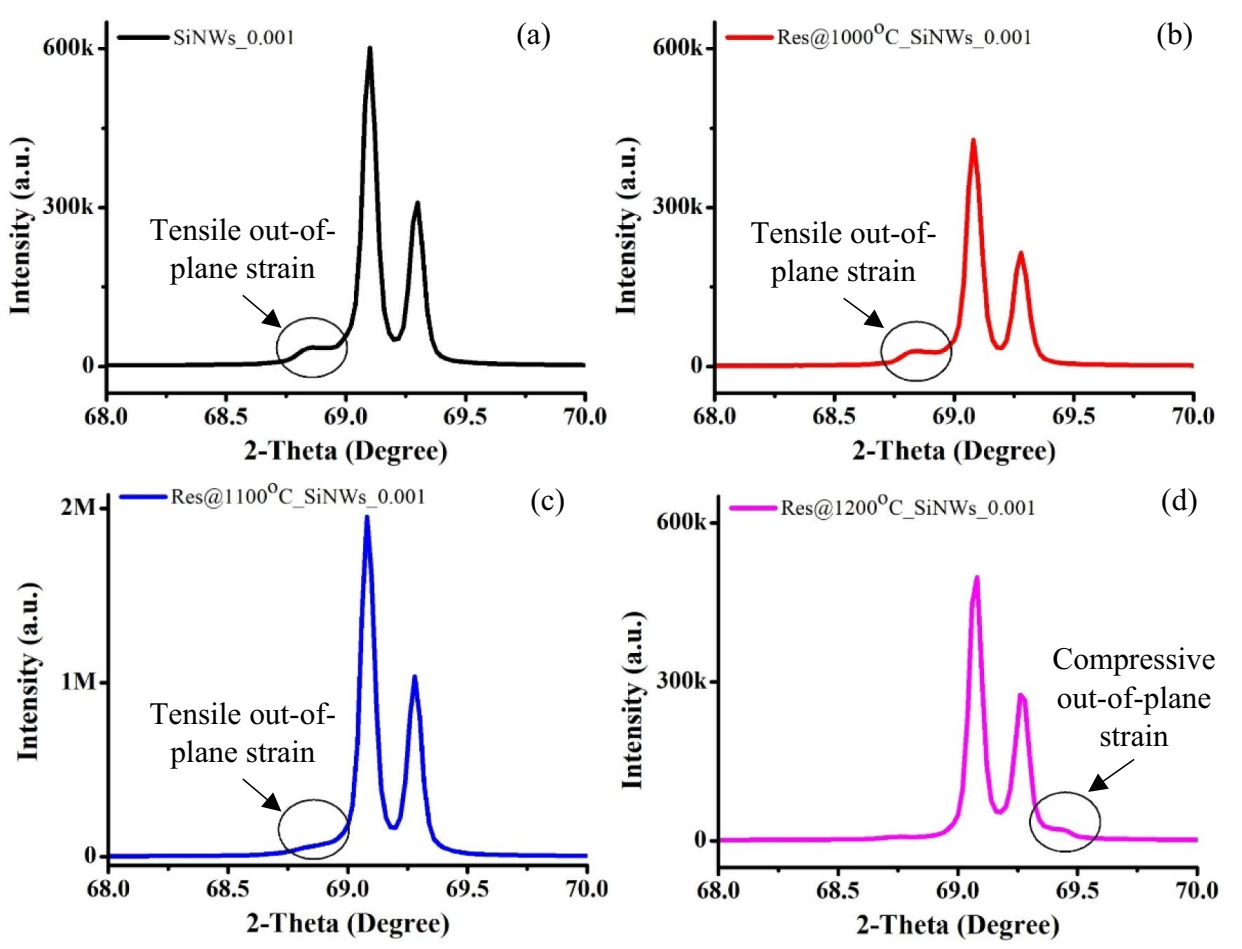
XRD pattern of pristine (**a**) and sintered SiNWs (**b**, **c**, **d**). A black color circle indicates the strain produced on the NWs. The tensile out-of-strain is highest in the pristine NWs, followed by sintered NWs (1000 °C). The tensile out-of-strain relaxes in sintered NWs (1100 °C). The tensile out-of-strain is converted to compressive out-of-strain for NWs sintered at 1200 °C.

Figure 9 shows the XRD pattern of pristine SiNWs and sintered SiNWs at various sintering temperatures. The highlighted circle indicates the formation of strain due to stress developed on the sidewalls of NWs array^39,40^. Out-of-plane lattice expansion in the pristine NWs produces tensile out-of-plane strain on the NWs, as shown in Fig. 9(a). The tensile out-of-plane strain remains for the NWs sintered at 1000 °C (Fig. 9(b)), whereas, at 1100 °C, the strain relaxes (Fig. 9(c)). However, at 1200 °C, the tensile out-of-plane strain is converted to compressive out-of-plane strain, as highlighted in Fig. 9(d).

**Figure 10:**
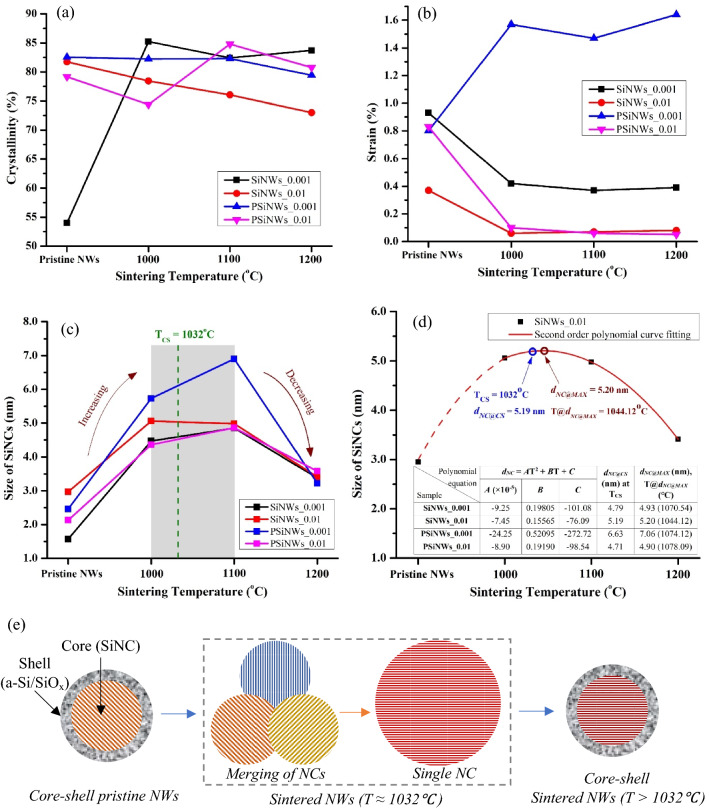
Parameters obtained from Raman characterization for pristine and sintered NWs such as (**a**) crystallinity, (**b**) strain, and (**c**) size of SiNCs (T_CS_ indicates critical sintering temperature). (**d**) second-order polynomial curve fitting for the size of SiNCs (*d*_*NC*_) with the sintering temperature (T) for SiNWs_0.01 sample. The table tabulates the fitting parameter values (*A*, *B*, and *C*) for all the sintered NWs, *d*_*NC@CS*_ at T_CS_, *d*_*NC@MAX*_, and corresponding sintering temperature T@*d*_*NC@MAX*_. (**e**) schematic of core–shell pristine NWs structure having smaller NC (T = 1000 °C), merging neighboring SiNCs to form a single larger NC at T ≈ 1032 °C, and formation of core–shell NWs having smaller NCs due to reoxidation at T > 1032 °C.

Figure 10 shows the changes in the morphological parameters of the pristine and sintered NWs array (crystallinity, strain, and size of Si nanostructures) obtained from the Raman spectra. The previous study^41^ reveals the synthesis of a core–shell NWs structure in the MACE, where the core contains c-Si nanostructures (e.g., quantum dots, nanocrystals), and the shell consists of a-Si phase. The tips of the NWs (in contact with the electrolyte) are etched rigorously upon MACE that destroys the periodic arrangement of atoms, i.e., the crystallinity of the NWs decreases. However, sintering melts the a-Si (present at the shell structure and the tips of the NWs), which decreases the diameter and length, and enhances crystallinity of the NWs. The upwards shift in the Raman peak of sintered NWs also confirms recrystallization in the Si atoms. Further, the sintering process rearranges the atoms in a periodic manner that increases the crystallinity, as shown in Fig. 10(a). Similarly, the pristine NWs exhibit higher strain^42^, and upon sintering, surface energy minimization stabilizes the lattice vibration and reduces stress and strain on the NWs array, as confirmed from Fig. 10(b). The Si nanostructures formed on the sidewalls of the NWs (due to secondary etching) are smaller in pristine NWs than the sintered NWs.

Upon sintering, the periodic rearrangement of atoms improves, increasing the average size of the Si nanocrystals (SiNCs) (i.e., *d*_*NC*_), as shown in Fig. 10(c). Holm et al.^25^ reported that sintering of Si nanoparticles merges differently oriented neighboring SiNCs at 750 °C (Fig. 10(e)), whereas the polycrystalline NP transforms to single-crystalline at 1000 °C. However, at 1200 °C, the NCs size decreases (i.e., after ~ T_CS_) because reoxidation destroys the periodic rearrangement, i.e., the crystallinity decreases due to the formation of a-Si/SiO_x_ shell on the core-SiNC. Figure 10(d) shows the parameters (*A*, *B*, and *C*) obtained after fitting the size of SiNCs with sintering temperature. The calculated *d*_*NC*_ at T_CS_ (i.e., *d*_*NC@CS*_), the maximum *d*_*NC*_ (i.e., *d*_*NC@MAX*_), and corresponding temperatures (i.e., T@*d*_*NC@MAX*_) are tabulated. The obtained size of the SiNCs (lesser than Bohr’s diameter) is responsible for producing QCE in the NWs array. Equation 1 represents an optimized second-order polynomial equation to calculate *d*_*NC*_ formed on the NWs due to sidewall etching for various sintering temperatures. The multiplying factor (*α*) and the error (*Ɛ*) are calculated and given in Table 1 for various NWs and resistivity of starting wafers.

1 $$ d_{NC} = \alpha \times \left( { - 1.25 \times 10^{ - 4} T^{2} + 0.26664T - 137.1075} \right) + 50 \times \varepsilon $$

**Table 1:** Multiplying factor (*α*) and error (*Ɛ*) for various sintered NWs sample.

#	SiNWs_0.001	SiNWs_0.01	PSiNWs_0.001	PSiNWs_0.01
*α*	0.7			
*Ɛ*	0.027	0.030	0.035	0.030

The XRD pattern also highlights the strain developed in the NWs array because of the out-of-plane lattice vibration. The NWs fabricated using lower resistivity Si/PSi substrate exhibit higher strain than the higher restive substrate. The strain also depends on the porosity of the sample, i.e., NWs fabricated using Si substrate exhibits lesser strain than the PSi substrate. Both Raman and XRD reveal the reduction of strain developed in the NWs after sintering. Removal of unstable Si atoms and nanograins of Si minimizes the surface energy of the sintered NWs. Therefore, sintering recrystallizes the NWs, reduces strain, and retains the QCE of the NWs. Sintering or heat-treatment tunes the morphology of the NWs as confirmed from the PL spectra, where the pristine NWs emit red light whereas the sintered NWs radiate blue and greenish-blue light. The integrated PL intensity confirms the reduction of electron–hole recombination in the sintered NWs, which is possible due to two reasons, i.e., (i) reduction of Ag remanence (removes the forbidden energy gap in between the valance band and conduction band), and (ii) reduction in surface oxidation in the sintered NWs array.

Sintering proves better than washing due to two significant advantages, i.e., (i) reduces the Ag remanence and (ii) improves the morphology of the NWs array simultaneously. After optimizing the sintering temperature (i.e., T_CS_), an optimization of sintering time and environment would further precise the sintering parameters to remove the Ag remanence and improve the structural attributes of SiNWs array.

## Conclusion

The defect center arises due to the remanence of AgNPs should be removed to improve the device performance. The surface tension of SiNWs disallows the liquid-based solution to penetrate inside the NWs array and remove the Ag particles. Removing the Ag particles through sintering is an alternative approach as wiping out is cumbersome from the superhydrophobic surface by a liquid-based solution. Sintering the NWs array above the melting temperature of the Ag particles (962 °C) reduces the remanence of the AgNPs, as confirmed from the SEM–EDS analysis. The XRD and TG-DSC analysis confirm oxides (SiO and Ag_2_O) in the NWs array due to the trace level of oxygen in the furnace, which reacts with the trapped AgNPs present inside the pores. The TG-DSC indicates the critical sintering temperature (T_CS_ = 1032 °C), around which a gradual change in chemical reaction occurs (endothermic reaction changes to an exothermic one). The % of mass loss is equivalent to the % of the mass gain for the NWs sintered at 1000 °C and 1100 °C. Therefore, the NWs sintered at 1000 °C and 1100 °C exhibit similar morphological parameters. However, the NWs sintered at 1200 °C exhibit a different morphological characteristic than the sintered sample at 1000 °C and 1100 °C.

The SEM–EDS characterization confirms a reduction of NWs length and Ag remanence upon sintering. The surface melting is responsible for the decrease in NWs length on sintering. Sintering produces new planes on the NWs array due to recrystallization of the NWs and solidification of the AgNPs. Sintering also reduces the unstable Si atoms, nanograins, surface defects, and surface oxidations from the NWs surface. The Raman spectra confirm the QCE in the pristine and sintered NWs array. Sintering enhances the surface properties by reducing strain and interfacial energy states and elevating crystallinities of the NWs array. An optimized equation is derived for calculating the size of the SiNCs formed on the NWs surface at various sintering temperatures. The neighboring SiNCs merge above 1000 °C to form a single NC, and the largest NCs form nearly at T_CS_, then the size decreases due to reoxidation at 1200 °C.

## Methods

### Fabrication of SiNWs and PSiNWs

The SiNWs array was fabricated using Si substrate (through Route—I) and PSiNWs array using PSi substrate (through Route—II), as shown in Fig. 1. In Route—I (step—I step—II), the Si wafer (*p*-type, boron-doped, < 100 > , 275 ± 25 μm thick) was cut into square shapes (1 cm × 1 cm) and cleaned by DI water, as shown in Fig. 1^20–22,39,41^. Two types of resistivity of the Si wafer, i.e., 0.01–0.02 Ω.cm and 0.001–0.005 Ω.cm, were considered to analyze the change in the NWs morphology. In Route—II (step—I step—I' step—II), the Si wafer undergoes anodization (HF: Ethanol = 1: 2, current density = 30 mA/cm^2^, time = 20 min) to fabricate PSi substrate, as shown in step—I' of Fig. 1^39,41^. The PSi substrate was cut into square shapes (1 cm × 1 cm) and cleaned with DI water. The cleaned Si or PSi substrate was dipped in an electrolyte, composed of 0.02 M AgNO_3_ (make: Sigma-Aldrich), 0.1765 M H_2_O_2_ (make: Fisher Scientific), and 4.8 M HF (make: Acros Organics), for 60 min at 25 °C, as shown in step—II^41^. Then the SiNWs or PSiNWs were washed with DI water, followed by HNO_3_ (30%) solution for a few minutes to remove Ag particles, as shown in step—III and step—IV. Dipping the NWs array in HF solution removes the oxide layer deposited on the surface during HNO_3_ treatment, as shown in step—V. The NWs were rinsed with DI water again to remove the AgNPs further, as shown in step—VI ^20,21,41^. Sintering is carried out in an Ar atmosphere to reduce the Ag remanence further, as shown in step—VII.

### Sintering/restructuring of SiNWs/PSiNWs

The large surface area of the NWs (porous sidewalls) attracted the native oxide to grow on the surface at an ambient atmosphere. To minimize the effect, the experiment should be carried out inside a closed environment. Sintering of NWs was carried out in argon (Ar) atmosphere (purity = 99.99%) for various sintering temperatures (i.e., 1000 °C, 1100 °C, and 1200 °C) at a fixed sintering time of 60 min inside an inhouse built tubular furnace. The furnace temperature was controlled by a PID controller, where the heating rate was kept at 5 °C/minute. After sintering, the furnace was cooled naturally, and the samples were taken out from the furnace and kept inside a desiccator. The experiment results in four pristine NWs samples (considering two types of substrates and two types of substrates resistivities) and twelve sintered NWs samples (each pristine sample sintered at three sintering temperatures). Each pristine NWs sample is named based on the resistivity of substrate and type of substrate used in the fabrication process, e.g., ‘SiNWs_0.001’ referred to the SiNWs fabricated using Si substrate having a resistivity of 0.001 Ω.cm. Each sintered NWs sample is named based on the pristine NWs sample and the sintering temperature, e.g., ‘Res@1000 °C_PSiNWs_0.01’ referred to the sintered PSiNWs (at 1000 °C) sample fabricated using PSi substrate having a resistivity of 0.01 Ω.cm.

### Characterizations

SEM–EDS (make: JEOL JSM- 6480 LV, Oxford instruments), Raman (make: Witech Germany Spectropro HRS 300 laser, Ag-ion source, 532 nm wavelength, 1800 grooves/mm grating), Photoluminescence (PL) (make: Witech Germany Spectropro HRS 300 laser, Ag-ion source, 325 nm exciton wavelength), XRD (make: Rigaku Ultima IV, *λ*_*Cu*_ = 0.154 nm, step size = 0.02°, 2*θ*: 20°-70°), and TG-DSC (make: Netzsch GmbH, STA 449F1 Jupiter, temperature range: 25 °C-1200 °C, heating rate 10 °C/minute, step size = 4 °C) characterizations were performed to visualize, quantify, and compare the Ag remanence and morphological parameters of the NWs array after sintering.

## Supplementary Information

Supplementary Information.
