# Supplementary material for: Removal of Ag remanence and improvement in structural attributes of silicon nanowires array via sintering
Source: Sci Rep. 2021 Dec 17;11:24189. doi: 10.1038/s41598-021-03654-5 (PMC8683431; doi:10.1038/s41598-021-03654-5)
Supplement: Supplementary file 1 — Supplementary Information. [file 41598_2021_3654_MOESM1_ESM.pdf]

Removal of Ag Remanence and Improvement in Structural Attributes of Silicon Nanowires Array via Sintering

***Paresh Kale[1], & Mihir Kumar Sahoo[2]**

X-ray Diffraction (XRD) analysis of pristine and sintered PSiNWs array. XRD analysis helps in determining the presence of AgNPs in the NWs array. Since the Ag particles oxidize quickly under atmospheric conditions, Ag_2O (220) and Ag_2O (222) planes are formed at the higher sintering temperature, as confirmed from supplementary Supplementary Figure **1**, which remains for all the sintered samples at 54.5°, 69.3°, respectively. The melted and evaporated Ag particles redeposit on the Si planes systematically in (200) and (111) orientations apart from (220). The most prominent XRD peak of AgNPs appears at 69.3° because of the (111) Si plane in the NWs' surface, as shown in supplementary Supplementary Figure **1** (c), and supplementary Supplementary Figure **1** (f).

While measuring the XRD spectra of pristine and sintered PSiNWs, the Si (111) plane remains for all the sintered samples, as shown in supplementary Supplementary Figure **1** (a), and prominent peaks appear for Res@1100°C_PSiNWs_0.01 sample, as given in supplementary Supplementary Figure **1** (d). As the doping level of starting Si/PSi substrates is high, only one plane, i.e., (111), is formed on the NWs surface apart from the (400) plane. A parallel growth orientation of Ag (111) was observed on Si (111) plane after MACE.

Supplementary Supplementary Figure **1** (a) shows a down peak shift of XRD spectra for sintered PSiNWs at 1100°C without any shift in other sintered NWs. Supplementary Supplementary Figure **1** (d) shows the disappearance of the Si (111) plane for sintered PSiNWs at 1200°C and a downshift for sintered SiNWs at 1000°C. However, the inset of supplementary Supplementary Figure **1** (d) shows the peak becomes prominent at 1100°C compared to other pristine and sintered NWs. The two XRD Si peaks (32.9° and 69.1°) shift downward for the sintered NWs at 1000°C, indicating decreased strain on the NWs. An upshift in the 1100°C sample indicates the relaxation of strain on the sintered NWs.

The XRD Si peaks of sintered NWs at 1100°C and 1200°C either retain their position or shift upward, indicating relaxed strain or decreased lattice vibration. The XRD peak shift confirms recrystallization and changes in lattice strain of the sintered NWs. The recrystallization produces dominant Si peaks upon sintering, as shown in supplementary Supplementary Figure **1**, i.e., the Si peak intensity increases. The small peak located at 61.6° is due to the residual Cu K_β (wavelength, $\lambda_{Cu\beta}$ = 0.139 nm) radiation to the Si (400) diffraction peak, as shown in supplementary Supplementary Figure **1** (b) and Supplementary Figure **1** (e).

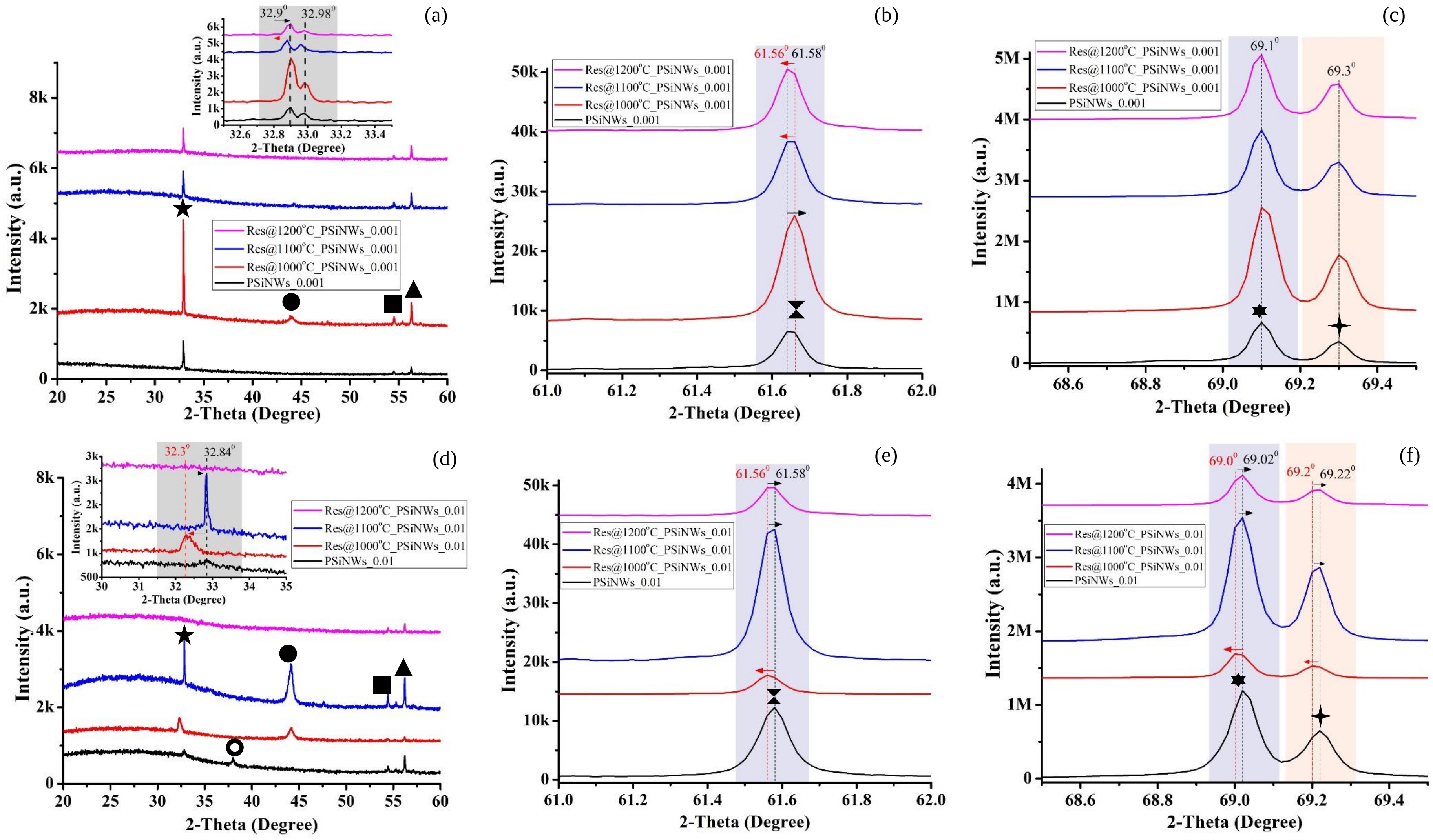

Supplementary Figure 1. XRD pattern of pristine and sintered (a, b, c) PSiNWs_0.001, (d, e, f) PSiNWs_0.01. The symbol represents ★(32.9°) → Si (111), ○(38.0°) → Ag (111), ●(44.2°) → Ag (200), ■(54.5°) → Ag_2O (220), ▲(56.3°) → Ag (220), ⧗(61.6°) → Si (400), ✶(69.1°) → Si (400), and ✦(69.3°) → Ag_2O (222).

Raman analysis of pristine and sintered SiNWs/PSiNWs array. Supplementary Supplementary Figure **2** shows higher FWHM of the NWs fabricated when using 0.001-0.005 Ω.cm resistivity as compared when using 0.01-0.02 Ω.cm resistivity of Si substrate, confirming the presence of higher amorphous content for lower resistivity substrate. Further, the amorphous content of NWs depends on the porosity of the sample, i.e., porous SiNWs (PSiNWs) exhibits higher FWHM compared to SiNWs sample, as shown in supplementary Supplementary Figure **2**. Asymmetrical broadening of FWHM indicates the formation of the Fano effect in the NWs. The Raman spectra of pristine NWs downshifts from the c-Si peak due to QCE as the secondary etching or sidewalls etching creates Si nanostructures or nanocrystals on the NWs.

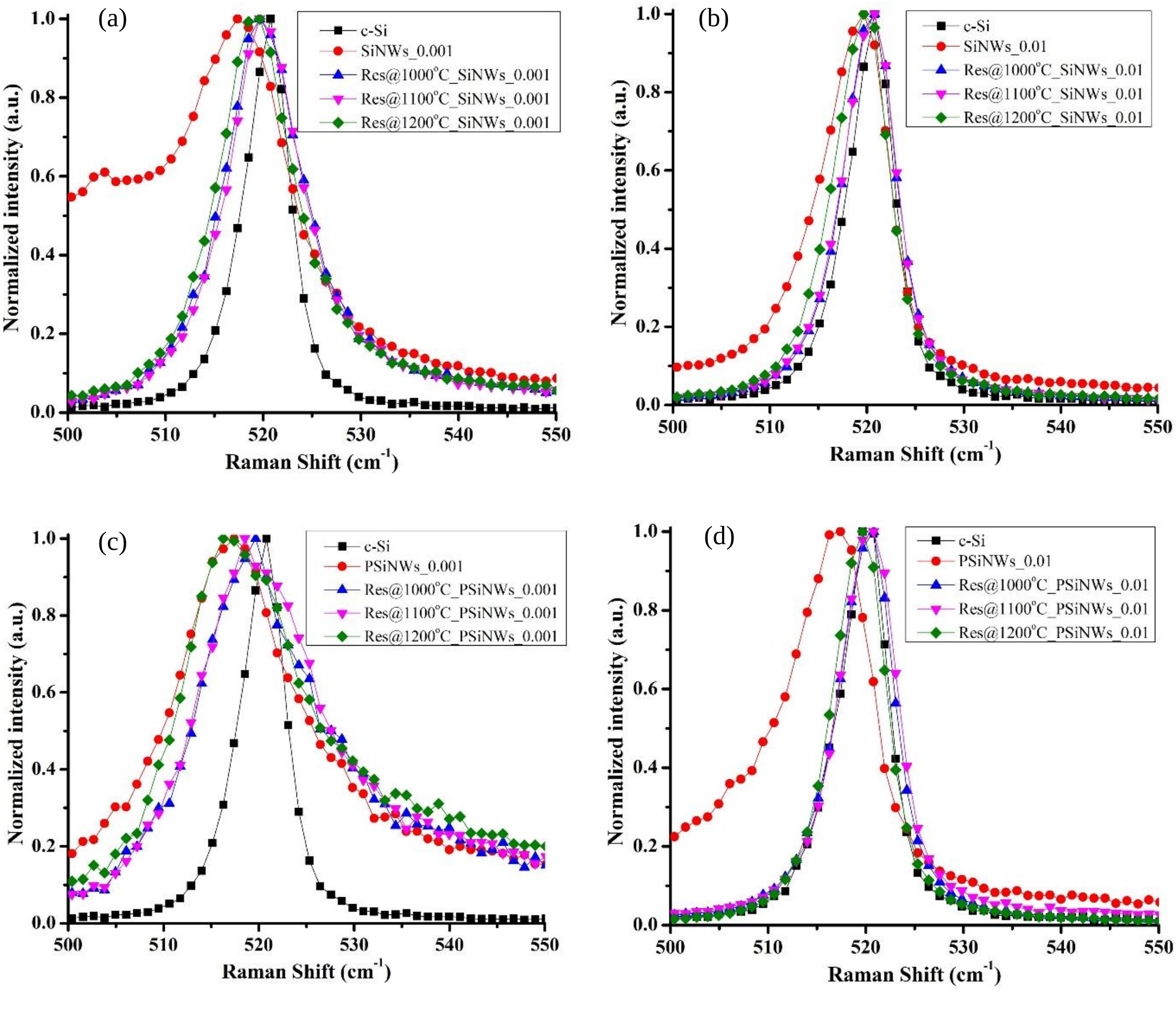

Supplementary Figure 2. Raman spectra of pristine and sintered NWs for (a) SiNWs_0.001, (b) SiNWs_0.01, (c) PSiNWs_0.001, (d) PSiNWs_0.01.

The FWHM of sintered NWs fabricated using lower resistive Si/PSi substrate (0.001-0.005 Ω.cm) broadens asymmetrically, confirming the retainment of the Fano effect, as shown in supplementary Supplementary Figure **2** (a) and supplementary Supplementary Figure **2** (c). However, the Fano effect

relaxes in the sintered NWs fabricated using higher resistive Si/PSi substrate (0.01-0.02 Ω.cm), i.e., FWHM narrows down, as shown in supplementary Supplementary Figure **2** (b) and supplementary Supplementary Figure **2** (d). After sintering, the Raman peak shifts towards the c-Si peak, confirming recrystallization. The Raman spectra of sintered samples at 1000°C and 1100°C look similar; however, the Raman peak downshifts again for the sintered sample at 1200°C compared to other samples confirming an increase in QCE. Therefore, the sintered NWs fabricated using a lower resistive substrate exhibit a FANTUM effect; however, the sintered NWs fabricated using a higher resistive substrate produce only the QCE.
